# Regulation of the *ACE2* locus in human airways cells

**DOI:** 10.1101/2020.10.04.325415

**Published:** 2020-11-06

**Authors:** Hye Kyung Lee, Olive Jung, Lothar Hennighausen

**Affiliations:** 1 Laboratory of Genetics and Physiology, National Institute of Diabetes, Digestive and Kidney Diseases, National Institutes of Health, Bethesda, Maryland 20892.; 2 Division of Preclinical Innovation, National Center for Advancing Translational Sciences, National Institutes of Health, Bethesda, Maryland 20892.

## Abstract

The angiotensin-converting enzyme 2 (ACE2) receptor is the gateway for SARS-CoV-2 to airway epithelium^1,2^ and the strong inflammatory response after viral infection is a hallmark in COVID-19 patients. Deciphering the regulation of the *ACE2* gene is paramount for understanding the cell tropism of SARS-CoV-2 infection. Here we identify candidate regulatory elements in the *ACE2* locus in human primary airway cells and lung tissue. Activating histone and promoter marks and Pol II loading characterize the intronic *dACE2* and define novel candidate enhancers distal to the genuine *ACE2* promoter and within additional introns. *dACE2*, and to a lesser extent *ACE2*, RNA levels increased in primary bronchial cells treated with interferons and this induction was mitigated by Janus kinase (JAK) inhibitors that are used therapeutically in COVID-19 patients. Our analyses provide insight into regulatory elements governing the *ACE2* locus and highlight that JAK inhibitors are suitable tools to suppress interferon-activated genetic programs in bronchial cells.

## Introduction

Recent studies^3,4^ have identified a novel short form of ACE2, called dACE2, that originates from an intronic promoter activated by interferons. Onabajo et al.^4^ used ENCODE data for chromatin modification marks (H3K4me3, H3K4me1 and H3K27ac) as well as DNase I hypersensitive (DHS) sites in cell lines to label putative regulatory elements at the newly identified exon (ex1c) located within intron 9 of the *ACE2* gene.

However, no regulatory elements were detected in the vicinity of the 5’ end of the full-length transcript encoding biologically active ACE2, and in sequences distal to the genuine promoter. Since these data sets were obtained from a wide range of cell lines and not from human primary airway cells, the principal target of SARS-CoV-2, they might not present a comprehensive picture of the regulatory regions controlling expression of the *dACE2* and the full-length *ACE2* transcripts in bronchial tissue.

## Results

**Figure 1.**
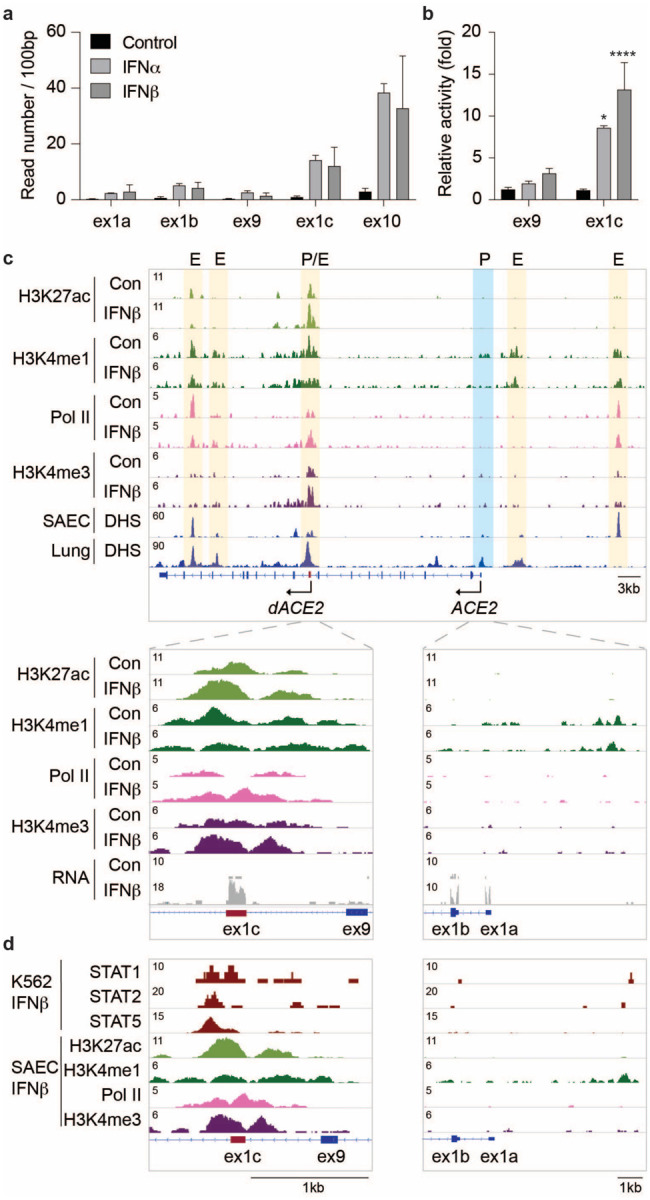
Regulation of the *ACE2* gene in primary airway epithelium. **a**. SAECs were cultured in the absence and presence of interferon alpha (IFNα) and beta (IFNβ) followed by RNA-seq analyses. The reads covering key exons (1a, 1b, 9, 1c and 10) are shown. **b.** mRNA levels of exon9 and exon1c were measured using qRT-PCR. Results are shown as the means ± s.e.m. of independent biological replicates (Control and IFNβ, *n* = 9; IFNα, *n* = 3). Two-way ANOVA with followed by Tukey’s multiple comparisons test was used to evaluate the statistical significance of differences. **c**. ChIP-seq experiments for the histone marks H3K4me3 (promoter), H3K4me1 (enhancers), H3K27ac (active genes) and Pol II loading. The DHS data were obtained from ENCODE^6,7^. Yellow shade, candidate enhancers and blue shade, predicted promoter. The P/E region within intron 9 probably constitutes a combined promoter/enhancer. **d**. Putative STAT5 enhancer in the *ACE2* gene was identified using ChIP-seq data from IFNβ treated K562 cells^8^.

To comprehensively identify the genetic elements controlling the extended *ACE2* locus, with an emphasis on its interferon response, we focused on human primary Small Airway Epithelial Cells (SAEC), which express a wide range of cytokine receptors and key mechanistic components of the executing JAK/STAT signal transduction pathway (Supplementary Table 1). We stimulated SAECs with interferon type I (IFNα and IFNβ), type II (IFNγ) and type III (IFNλ) as well as with growth hormone (GH), Interleukin 6 (IL6) and IL7, followed by RNA-seq transcriptome analyses (Supplementary Tables 2–8). Increased *ACE2* expression was obtained with the interferons but not with GH, IL6 and IL7 (Supplementary Figure 1). However, the induction was less than that seen for classical interferon stimulated genes (ISG), such as *STAT1*. In agreement with earlier studies^3,4^, we detected the novel *dACE2* N-terminal exon (ex1c) within intron 9 of the *ACE2* gene (Supplementary Figure 1). To obtain more definitive information on the interferon response of the *dACE2* and *ACE2* promoters, we used RNA-seq and determined the respective read counts over the three alternative first exons (Figure 1a and Supplementary Figure 1d). While the increase of RNA-seq reads induced by IFNα/β was highest (~25-fold) over ex1c, a lesser, yet significant, ~2–10-fold increase was detected over ex1a and ex1b, supporting the notion that expression of the full-length *ACE2* transcript is also under interferon control. As an independent assay we used qRT-PCR and determined that IFN α/β stimulation led to a 8 to 15-fold increase of *dACE2* and an approximately ~3-fold increase of ACE2 RNA (Figure 1b). However, the degree of induction of either form was lower than that seen for *bona fide* ISGs (Supplementary Figure 1). Previous studies in normal human bronchial epithelium (NHBE) did not reveal an interferon response of the native *ACE2* promoter^3,4^ suggesting differences between cell types or growth conditions. The mouse *Ace2* gene is induced by cytokines through a STAT5-based enhancer in the second intron^5^ and a DHS site is located in the equivalent location in the human *ACE2* gene in SAEC and lung tissue. This suggests the presence of additional regulatory elements controlling expression of the full-length *ACE2* mRNA.

To identify candidate regulatory elements controlling the extended *ACE2* locus, including *ACE2* and *dACE2*, in primary airway cells, we dug deeper and conducted ChIP-seq for the chromatin marks H3K27ac (activate loci), H3K4me1 (enhancers), H3K4me3 (promoters) and Polymerase II (Pol II) loading, both in the absence and presence of IFNβ (Figure 1c Supplementary Figure 2). DNase I hypersensitive (DHS) sites from lung tissues^6^ and SAECs^7^ served as *bona fide* predictors of regulatory regions. In addition to *ACE2*, the neighboring *TMEM27* gene, an *ACE2* homologue, as well as *BMX* are activated by interferons (Supplementary Figure 2) suggesting the possibility of jointly used regulatory elements. *ACE2* and *TMEM27* originated from a gene duplication and their response to interferon is equivalent. The positions of the chromatin boundary factor CTCF suggests that *ACE2* and *TMEM27* are located within a sub-TAD (Supplementary Figure 2).

In agreement with earlier studies^4^, we identified DHS sites at ex1c and in intron 17 (Figure 1c). In addition, we identified DHS sites at ex1a, likely marking the genuine *ACE2* promoter, a distal site marking a possible enhancer and in intron 15 coinciding with activate marks. The DHS site in intron 9 overlaps with strong H3K4me3 marks, identifying it as a genuine promoter region (Figure 1c). This site is also decorated with H3K4me1 and H3K27ac marks and extensive Pol II loading, hallmarks of a complex promoter/enhancer. H3K27ac and Pol II loading was further induced by IFNβ, reflecting increased *ACE2* expression. IFNβ activates the transcription factors STATs 1, 2 and 5 and ChIP-seq experiments from K562 erythroid cells stimulated with IFNβ^8^ revealed preferential binding of STAT5 to the intronic promoter/enhancer (Figure 1d) further supporting regulation through the JAK/STAT pathway. The STAT1 locus served as a control for the binding of STAT transcription factors (Supplementary Figure 3). While the *ACE2* promoter associated with ex1a is marked by a DHS site and H3K4me1 marks, there is little evidence of H3K4me3 and H3K27ac marks. However, it is well known that there is no direct relationship between gene activity and the presence of these marks.

Based on the presence of DHS sites, activating chromatin marks and Pol II loading, either in combination or by themselves, we predict additional enhancers. A prominent candidate enhancer, marked by a DHS, H3K4me1 and Pol II loading is positioned approximately 16 kb distal of ex1a and additional putative regulatory regions are located with introns (Figure 1c). Activating histone marks and DHS sites^6,7^ are also present in the genuine *ACE2* promoter and the distal region in lung tissue (Supplementary Figure 4).

**Figure 2.**
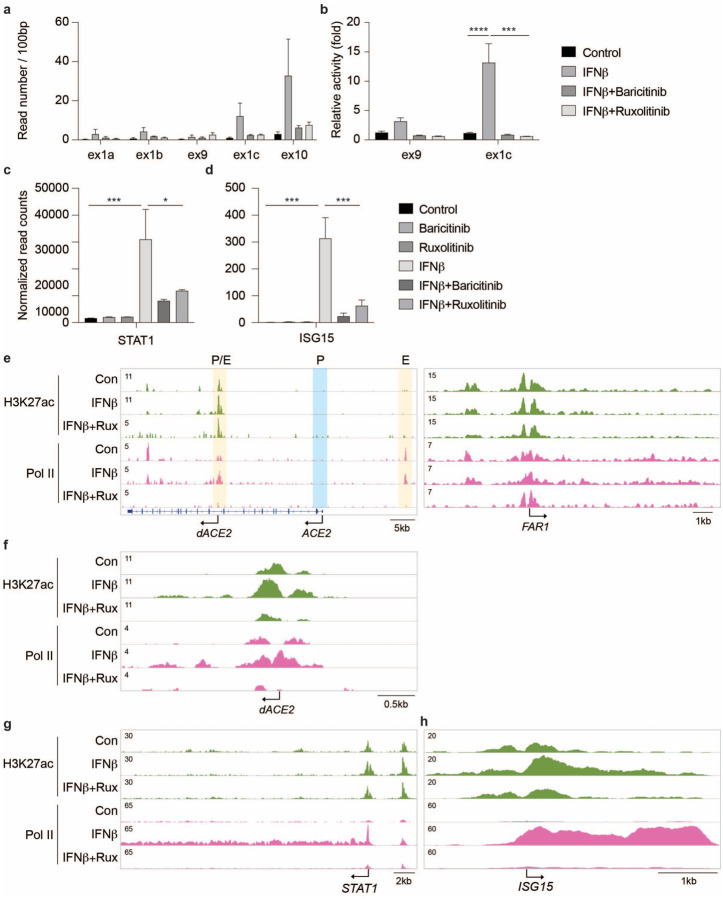
JAK inhibitors mitigated activation of IFNβ-stimulated genes. **a-b.** SAECs were cultured in the presence of IFNβ and JAK inhibitors, either Baricitinib or Ruxolitinib followed by RNA-seq analyses and qRT-PCR. Reads covering key exons are displayed. qRT-PCR results are shown as the means ± s.e.m. of independent biological replicates (Control and IFNβ, *n* = 9; IFNβ+JAK inhibitors, *n* = 3). Two-way ANOVA with followed by Tukey’s multiple comparisons test was used to evaluate the statistical significance of differences. **c-d.** *STAT1* and *ISG15* mRNA levels from control and experimental cells were measured by RNA-seq. Results are shown as the means ± s.e.m. of independent biological replicates (*n* = 3). One-way ANOVA with followed by Dunnett’s multiple comparisons test was used to evaluate the statistical significance of differences. **e-h.** H3K27ac marks, and Pol II loading at the *ACE2*, *STAT1* and *ISG15* loci in SAECs in the absence and presence of IFNβ and the JAK inhibitor, Ruxolitinib.

Interferons activate genetic programs through the JAK/STAT signaling pathway and JAK inhibitors are used clinically in COVID-19 patients in an effort to suppress the genomic consequences of cytokine storms^9,10,11^. To investigate if interferon-induced *ACE2* expression is controlled by the JAK/STAT pathway, we cultured SAECs in the presence of IFNβ and the JAK inhibitors, Baricitinib and Ruxolitinib, followed by RNA-seq and qRT-PCR assays (Supplementary Tables 9–10 and Figure 2) and ChIP-seq analyses (Figure 2). Both inhibitors suppressed the IFNβ-induced increase of the full-length *ACE2* (ex1a, ex1b and ex9) and the *dACE2* (ex1c) transcripts (Figure 2a and b), supporting that their respective promoters are under JAK/STAT control. The efficacy of the two inhibitors extended to a range of genetic programs activated through the pan JAK/STAT pathway (Figure 2c and Supplementary Figure 5) and induction of *bona fide* interferon stimulated genes (ISG), such as *ISG15*, was suppressed (Figure 2d). In *ACE2*, as in other ISGs, Ruxolitinib treatment mitigated the establishment of activating H3K27ac marks and Pol II loading over distal and intronic regulatory elements (Figure 2e–h).

## Discussion

In summary, we have assessed the extended *ACE2* locus for regulatory elements controlling gene expression induced by interferons and other, not yet defined, stimuli. We show that the intronic regulatory region bears promoter and enhancer marks and binds STAT transcription factors, thus constituting a complex regulatory element controlling interferon-induced *dACE2* expression^4^. We identified additional candidate regulatory elements throughout the locus that can be invoked in controlling transcription of the full-length transcripts yielding the biologically active ACE2. Regulation of the human and mouse *ACE2*^5^ loci displays distinct differences, yet they share their response to cytokines and the JAK/STAT pathway and the mouse *Ace2* gene is activated in mammary tissue by cytokines through a JAK2/STAT5-dependent intronic enhancer^5^. Further studies in tissues and primary cells are needed to understand the complex, possibly cell-specific, regulation of the human *ACE2* locus in organs with extrapulmonary manifestations of SARS-CoV-2 infection. Our demonstration that JAK inhibitors mitigate interferon-induced activation of inflammatory programs has practical implications for their use in treating COVID-19 patients.

## Supplementary Material

1
