# Supplementary material for: Regulation of the ACE2 locus in human airways cells
Source: bioRxiv. 2020 Nov 6:2020.10.04.325415. Originally published 2020 Oct 5. Preprint. [Version 2] doi: 10.1101/2020.10.04.325415 (PMC7553184; doi:10.1101/2020.10.04.325415)
Supplement: 1 [file NIHPP2020.10.04.325415-supplement-1.pdf]

bioRxiv preprint doi: https://doi.org/10.1101/2020.10.04.325415; this version posted November 6, 2020. The copyright holder for this preprint (which was not certified by peer review) is the author/funder. This article is a US Government work. It is not subject to copyright under 17 USC 105 and is also made available for use under a CC0 license.

Supplementary Information

Methods

Cell culture

Human small airway epithelial cells (SAEC) obtained from Lifeline Technology (FC-0016) were expanded using the complete BronchiaLife™ media kit (Lifeline Technology, LL-0023). All culture wares were pre-coated in 30 μg/ml of Fibronectin (ThermoFisher Scientific, 33016015) for at least 1h at room temperature. Calu-3 line (ATCC, HTB-55™) were cultured using Eagle's Minimum Essential Medium (ATCC, 30-2003™) containing 10% fetal bovine serum (Cytiva, SH3007103) in 5% CO2 atmosphere at 37°C.

Cytokines (10 ng/ml; Human IFNβ, 300-02BC; Human IFNγ, 300-02; Human IL6, 200-06; Human IL7, 200-07; Human Growth hormone, 100-40, Peprotech; Human IFNα2b, 78077.1, Stem Cell Technologies; Human IFNλ3, 5259-IL-025, R&D systems) were treated in respective culture media and the cells were incubated for 12hr in 5% CO2 atmosphere at 37°C. The cells were washed with PBS (Gibco, 14190144) twice and harvested.

Jak inhibitors, 10 μM of either Baricitinib (HY-15315A, MedChemExpress) or Ruxolitinib (HY-50856A, MedChemExpress), were added to BronchiLiafe™ media with or without IFNβ. SAEC and incubated for 12hrs and then washed with PBS twice and harvested.

RNA isolation and quantitative real-time PCR (qRT–PCR)

Total RNA was extracted from the collected cells and purified using the PureLink RNA Mini Kit (Invitrogen) according to the manufacturer's instructions. cDNA was synthesized from total RNA using Superscript II (Invitrogen). Quantitative real-time PCR (qRT-PCR) was performed using TaqMan probes (ACE2, Hs01085333_m1; *STAT1*, Hs01013996_m1; *GAPDH*, Hs02786624_g1, Thermo Fisher scientific) on the CFX384 Real-Time PCR Detection System (Bio-Rad) according to the manufacturer's instructions. Exon9 and exon1c mRNA were measured with the following primers, that were used by Onabajo et al.[4], using SYBR Green system: Forward, 5'-GGGCGACTTCAGGATCCTTAT-3', Reverse, 5'-GGATATGCCCCATCTCATGATGG-3';

bioRxiv preprint doi: https://doi.org/10.1101/2020.10.04.325415; this version posted November 6, 2020. The copyright holder for this preprint (which was not certified by peer review) is the author/funder. This article is a US Government work. It is not subject to copyright under 17 USC 105 and is also made available for use under a CC0 license.

Forward, 5’-GGAAGCAGGCTGGGACAAA-3’, Reverse, 5’-AGCTGTCAGGAAGTCGTCCATTG-3’. PCR conditions were 95°C for 30s, 95°C for 15s, and 60°C for 30s for 40 cycles. All reactions were done in triplicate and normalized to the housekeeping gene *GAPDH*. Relative differences in PCR results were calculated using the comparative cycle threshold (C_T) method.

Total RNA sequencing (Total RNA-seq) and data analysis.

Total RNA was extracted from the collected cells and purified using the PureLink RNA Mini Kit (Invitrogen) according to the manufacturer’s instructions. Ribosomal RNA was removed from 1 µg of total RNAs and cDNA was synthesized using SuperScript III (Invitrogen). Libraries for sequencing were prepared according to the manufacturer’s instructions with TruSeq Stranded Total RNA Library Prep Kit with Ribo-Zero Gold (Illumina, RS-122-2301) and paired-end sequencing was done with a HiSeq 3000 instrument (Illumina).

Total RNA-seq read quality control was done using Trimmomatic[12] (version 0.36) and STAR RNA-seq[13] (version STAR 2.5.4a) using 50bp paired-end mode was used to align the reads (hg19). HTSeq[14] was to retrieve the raw counts and subsequently, R (https://www.R-project.org/), Bioconductor[15] and DESeq2[16] were used. Additionally, the RUVSeq[17] package was applied to remove confounding factors. The data were pre-filtered keeping only those genes, which have at least ten reads in total. Genes were categorized as significantly differentially expressed with an adjusted p-value (pAdj) below 0.05 and a fold change > 2 for up-regulated genes and a fold change of < -2 for down-regulated ones. The visualization was done using dplyr (https://CRAN.R-project.org/package=dplyr) and ggplot2[18]. Sequence read numbers were calculated using Samtools[19] software with sorted bam files.

Chromatin immunoprecipitation sequencing (ChIP-seq) and data analysis.

Chromatin was fixed with formaldehyde (1% final concentration) for 15 min at room temperature, and then quenched with glycine (0.125 M final concentration). Samples were processed as previously described[20]. The following antibodies were used for ChIP-seq: H3K27ac (Abcam, ab4729), RNA polymerase II (Abcam, ab5408), H3K4me1 (Active

bioRxiv preprint doi: https://doi.org/10.1101/2020.10.04.325415; this version posted November 6, 2020. The copyright holder for this preprint (which was not certified by peer review) is the author/funder. This article is a US Government work. It is not subject to copyright under 17 USC 105 and is also made available for use under a CC0 license.

Motif, 39297) and H3K4me3 (Millipore, 07-473). Libraries for next-generation sequencing were prepared and sequenced with a HiSeq 3000 instrument (Illumina). Quality filtering and alignment of the raw reads was done using Trimmomatic[12] (version 0.36) and Bowtie[21] (version 1.1.2), with the parameter '-m 1' to keep only uniquely mapped reads, using the reference genome hg19. Picard tools (Broad Institute. Picard, http://broadinstitute.github.io/picard/. 2016) was used to remove duplicates and subsequently, Homer[22] (version 4.8.2) software was applied to generate bedGraph files. Integrative Genomics Viewer[23] (version 2.3.81) was used for visualization.

Statistical anlaysis

For comparison of samples, data were presented as standard deviation in each group and were evaluated with a two-way ANOVA followed by Tukey's multiple comparisons test or a one-way ANOVA with Dunnett's multiple comparisons test using PRISM 8 GraphPad (version 8.2.0). Statistical significance was obtained by comparing the measures from wild-type or control group, and each mutant group. A value of $*P < 0.05$, $**P < 0.001$, $***P < 0.0001$, $****P < 0.00001$ was considered statistically significant.

Data availability. All data were obtained or uploaded to Gene Expression Omnibus (GEO). ChIP-seq for STATs was obtained under GSE31477. ChIP-seq data for H3K27ac and H3K4me1 from human lung tissues were downloaded from GSE143115 and 142958. DNase I hypersensitive (DHS) data from human lung tissues and SAEC were obtained under GSE90364 and 29692, respectively. The RNA-seq and ChIP-seq data from SAEC will be uploaded in GEO before publishing the manuscript.

Acknowledgments

We thank Ilhan Akan, Sijung Yun and Harold Smith from the NIDDK genomics core for NGS and NCATS Chemical Genomics Center Team for the JAK inhibitors. This work utilized the computational resources of the NIH HPC Biowulf cluster (http://hpc.nih.gov).

Funding

bioRxiv preprint doi: https://doi.org/10.1101/2020.10.04.325415; this version posted November 6, 2020. The copyright holder for this preprint (which was not certified by peer review) is the author/funder. This article is a US Government work. It is not subject to copyright under 17 USC 105 and is also made available for use under a CC0 license.

This work was supported by the Intramural Research Programs (IRPs) of National Institute of Diabetes and Digestive and Kidney Diseases (NIDDK) and National Center for Advancing Translational Sciences (NCATS).

Author contribution

HKL: project conception, experimental design and execution, data analysis, preparation of figures, writing manuscript; OJ: experimental design and execution; LH: project conception, experimental design, data analysis, preparation of figures, writing manuscript.

Competing interests

The authors declare no competing financial interests.

References

1. Lukassen, S. *et al.* SARS-CoV-2 receptor ACE2 and TMPRSS2 are primarily expressed in bronchial transient secretory cells. *EMBO J*, e105114 (2020).
2. Hoffmann, M. *et al.* SARS-CoV-2 Cell Entry Depends on ACE2 and TMPRSS2 and Is Blocked by a Clinically Proven Protease Inhibitor. *Cell* **181**, 271-280 e8 (2020).
3. Ng, K.W. *et al.* Tissue-specific and interferon-inducible expression of nonfunctional ACE2 through endogenous retroelement co-option. *Nat Genet* (2020).
4. Onabajo, O.O. *et al.* Interferons and viruses induce a novel truncated ACE2 isoform and not the full-length SARS-CoV-2 receptor. *Nat Genet* (2020).
5. Hennighausen, L. & Lee, H.K. Activation of the SARS-CoV-2 Receptor Ace2 through JAK/STAT-Dependent Enhancers during Pregnancy. *Cell Rep*, 108199 (2020).
6. Consortium, E.P. An integrated encyclopedia of DNA elements in the human genome. *Nature* **489**, 57-74 (2012).
7. Maurano, M.T. *et al.* Large-scale identification of sequence variants influencing human transcription factor occupancy in vivo. *Nat Genet* **47**, 1393-401 (2015).

bioRxiv preprint doi: https://doi.org/10.1101/2020.10.04.325415; this version posted November 6, 2020. The copyright holder for this preprint (which was not certified by peer review) is the author/funder. This article is a US Government work. It is not subject to copyright under 17 USC 105 and is also made available for use under a CC0 license.

8. Au-Yeung, N., Mandhana, R. & Horvath, C.M. Transcriptional regulation by STAT1 and STAT2 in the interferon JAK-STAT pathway. *JAKSTAT* **2**, e23931 (2013).
9. La Rosee, F. *et al.* The Janus kinase 1/2 inhibitor ruxolitinib in COVID-19 with severe systemic hyperinflammation. *Leukemia* **34**, 1805-1815 (2020).
10. Yeleswaram, S. *et al.* Inhibition of cytokine signaling by ruxolitinib and implications for COVID-19 treatment. *Clin Immunol* **218**, 108517 (2020).
11. Kinase Inhibitors: Bruton's Tyrosine Kinase Inhibitors and Janus Kinase Inhibitors. (https://www.covid19treatmentguidelines.nih.gov/immune-based-therapy/immunomodulators/kinase-inhibitors/)
12. Bolger, A.M., Lohse, M. & Usadel, B. Trimmomatic: a flexible trimmer for Illumina sequence data. *Bioinformatics* **30**, 2114-20 (2014).
13. Dobin, A. *et al.* STAR: ultrafast universal RNA-seq aligner. *Bioinformatics* **29**, 15-21 (2013).
14. Anders, S., Pyl, P.T. & Huber, W. HTSeq--a Python framework to work with high-throughput sequencing data. *Bioinformatics* **31**, 166-9 (2015).
15. Huber, W. *et al.* Orchestrating high-throughput genomic analysis with Bioconductor. *Nat Methods* **12**, 115-21 (2015).
16. Love, M.I., Huber, W. & Anders, S. Moderated estimation of fold change and dispersion for RNA-seq data with DESeq2. *Genome Biol* **15**, 550 (2014).
17. Risso, D., Ngai, J., Speed, T.P. & Dudoit, S. Normalization of RNA-seq data using factor analysis of control genes or samples. *Nat Biotechnol* **32**, 896-902 (2014).
18. Wickham, H. *Ggplot2 : elegant graphics for data analysis*, viii, 212 p. (Springer, New York, 2009).
19. Masella, A.P. *et al.* BAMQL: a query language for extracting reads from BAM files. *BMC Bioinformatics* **17**, 305 (2016).
20. Metser, G. *et al.* An autoregulatory enhancer controls mammary-specific STAT5 functions. *Nucleic Acids Res* **44**, 1052-63 (2016).

bioRxiv preprint doi: https://doi.org/10.1101/2020.10.04.325415; this version posted November 6, 2020. The copyright holder for this preprint (which was not certified by peer review) is the author/funder. This article is a US Government work. It is not subject to copyright under 17 USC 105 and is also made available for use under a CC0 license.

21. Langmead, B., Trapnell, C., Pop, M. & Salzberg, S.L. Ultrafast and memory-efficient alignment of short DNA sequences to the human genome. *Genome Biol* **10**, R25 (2009).

22. Heinz, S. *et al.* Simple combinations of lineage-determining transcription factors prime cis-regulatory elements required for macrophage and B cell identities. *Mol Cell* **38**, 576-89 (2010).

23. Thorvaldsdottir, H., Robinson, J.T. & Mesirov, J.P. Integrative Genomics Viewer (IGV): high-performance genomics data visualization and exploration. *Brief Bioinform* **14**, 178-92 (2013).

bioRxiv preprint doi: https://doi.org/10.1101/2020.10.04.325415; this version posted November 6, 2020. The copyright holder for this preprint (which was not certified by peer review) is the author/funder. This article is a US Government work. It is not subject to copyright under 17 USC 105 and is also made available for use under a CC0 license.

Supplementary Table 1. mRNA levels of genes associated with the pan JAK-STAT pathway in SAEC.

Supplementary Table 2. List of all genes with normalized read counts in each replicate at Control and IFNα treated SAEC, $\log_2$ (fold change), *p*-value and adjusted *p*-value as well as upregulated gene list and GSEA analysis.

Supplementary Table 3. List of all genes with normalized read counts in each replicate at Control and IFNβ treated SAEC, $\log_2$ (fold change), *p*-value and adjusted *p*-value as well as upregulated gene list and GSEA analysis.

Supplementary Table 4. List of all genes with normalized read counts in each replicate at Control and IFNγ treated SAEC, $\log_2$ (fold change), *p*-value and adjusted *p*-value as well as upregulated gene list and GSEA analysis.

Supplementary Table 5. List of all genes with normalized read counts in each replicate at Control and IFNλ3 treated SAEC, $\log_2$ (fold change), *p*-value and adjusted *p*-value as well as upregulated gene list and GSEA analysis.

Supplementary Table 6. List of all genes with normalized read counts in each replicate at Control and IL6 treated SAEC, $\log_2$ (fold change), *p*-value and adjusted *p*-value as well as upregulated gene list and GSEA analysis.

Supplementary Table 7. List of all genes with normalized read counts in each replicate at Control and IL7 treated SAEC, $\log_2$ (fold change), *p*-value and adjusted *p*-value as well as upregulated gene list and GSEA analysis.

Supplementary Table 8. List of all genes with normalized read counts in each replicate at Control and GH treated SAEC, $\log_2$ (fold change), *p*-value and adjusted *p*-value as well as upregulated gene list and GSEA analysis.

bioRxiv preprint doi: https://doi.org/10.1101/2020.10.04.325415; this version posted November 6, 2020. The copyright holder for this preprint (which was not certified by peer review) is the author/funder. This article is a US Government work. It is not subject to copyright under 17 USC 105 and is also made available for use under a CC0 license.

Supplementary Table 9. List of all genes with normalized read counts in each replicate at IFNβ and Baricitinib with IFNβ, treated SAEC, $\log_2$ (fold change), *p*-value and adjusted *p*-value as well as upregulated gene list and GSEA analysis.

Supplementary Table 10. List of all genes with normalized read counts in each replicate at IFNβ and Ruxolitinib with IFNβ, treated SAEC, $\log_2$ (fold change), *p*-value and adjusted *p*-value as well as upregulated gene list and GSEA analysis.

bioRxiv preprint doi: https://doi.org/10.1101/2020.10.04.325415; this version posted November 6, 2020. The copyright holder for this preprint (which was not certified by peer review) is the author/funder. This article is a US Government work. It is not subject to copyright under 17 USC 105 and is also made available for use under a CC0 license.

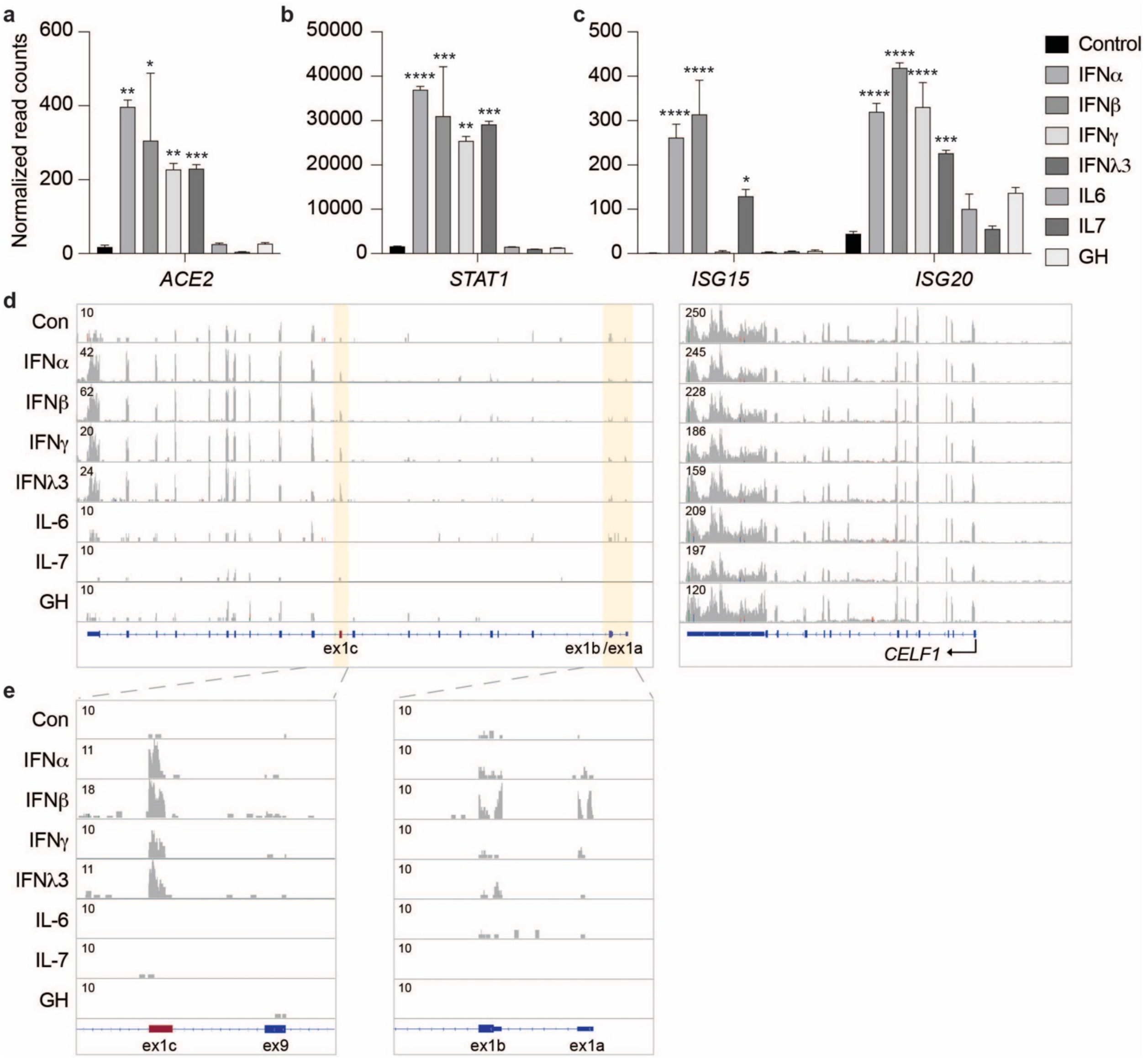

Supplementary Figure 1. Induction of *ACE2* expression by interferons. a-c. *ACE2*, and *STAT1* and *ISGs* mRNA levels from control cells and cells treated with different cytokines were measured by RNA-seq. Results are shown as the means ± s.e.m. of independent biological replicates (n = 3). One-way ANOVA followed by Dunnett's multiple comparisons test was used to evaluate the statistical significance of differences. **d-e.** RNA-seq reads are matched with exons.

bioRxiv preprint doi: https://doi.org/10.1101/2020.10.04.325415; this version posted November 6, 2020. The copyright holder for this preprint (which was not certified by peer review) is the author/funder. This article is a US Government work. It is not subject to copyright under 17 USC 105 and is also made available for use under a CC0 license.

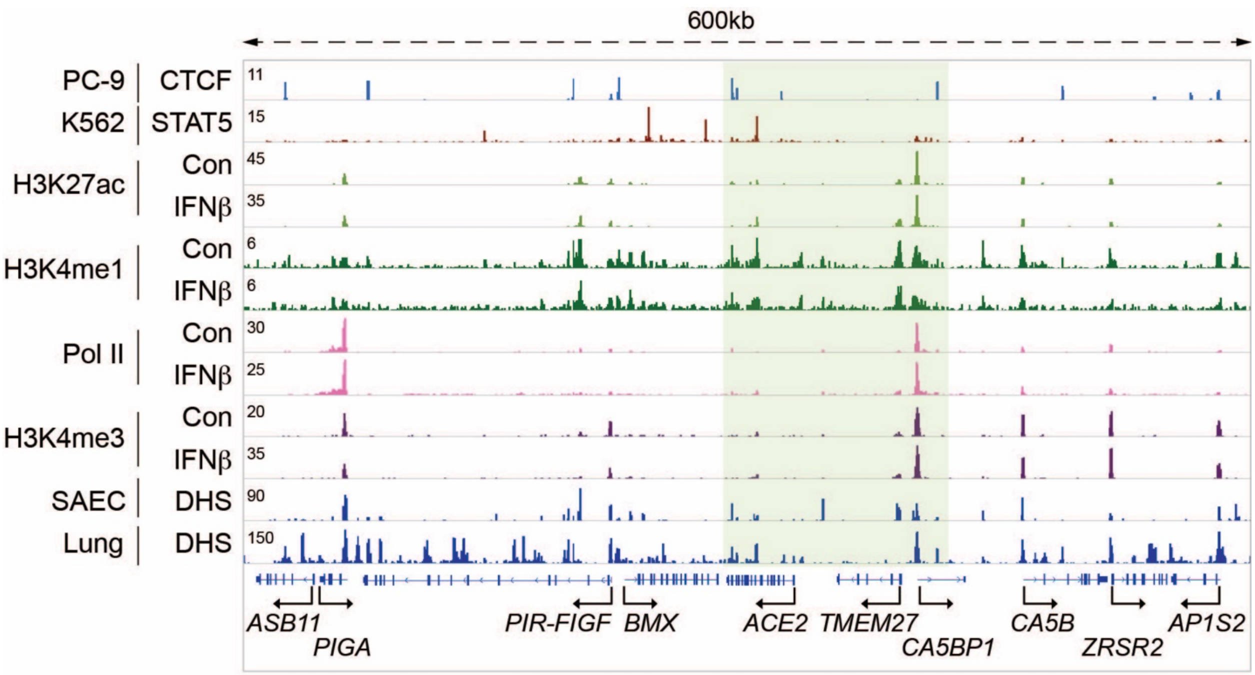

RNA-seq	*PIGA*	*PIR-FIGF*	*BMX*	*ACE2*	*TMEM27*	*CA5BP1*	*CA5B*
Con	493.8	1.4	2.0	17.2	9.0	31.6	109.1
IFNβ	673.3	1.0	13.1	305.0	207.7	59.7	153.8
Induction	1.4	0.8	6.4	17.7	23.1	1.9	1.4

Supplementary Figure 2. Structure and expression of the extended ACE2 locus in primary airway cells. Regulatory marks in the 600 kb locus including *ACE2* and neighboring genes in SAECs. mRNA levels in the absence and presence of IFNβ were measured by RNA-seq. ChIP-seq for H3K4me1, H3K27ac, H3K4me3 and Pol II was conducted in SAECs in the absence and presence of IFNβ. CTCF and STAT5 ChIP-seq data and DHS data are from ENCODE.

bioRxiv preprint doi: https://doi.org/10.1101/2020.10.04.325415; this version posted November 6, 2020. The copyright holder for this preprint (which was not certified by peer review) is the author/funder. This article is a US Government work. It is not subject to copyright under 17 USC 105 and is also made available for use under a CC0 license.

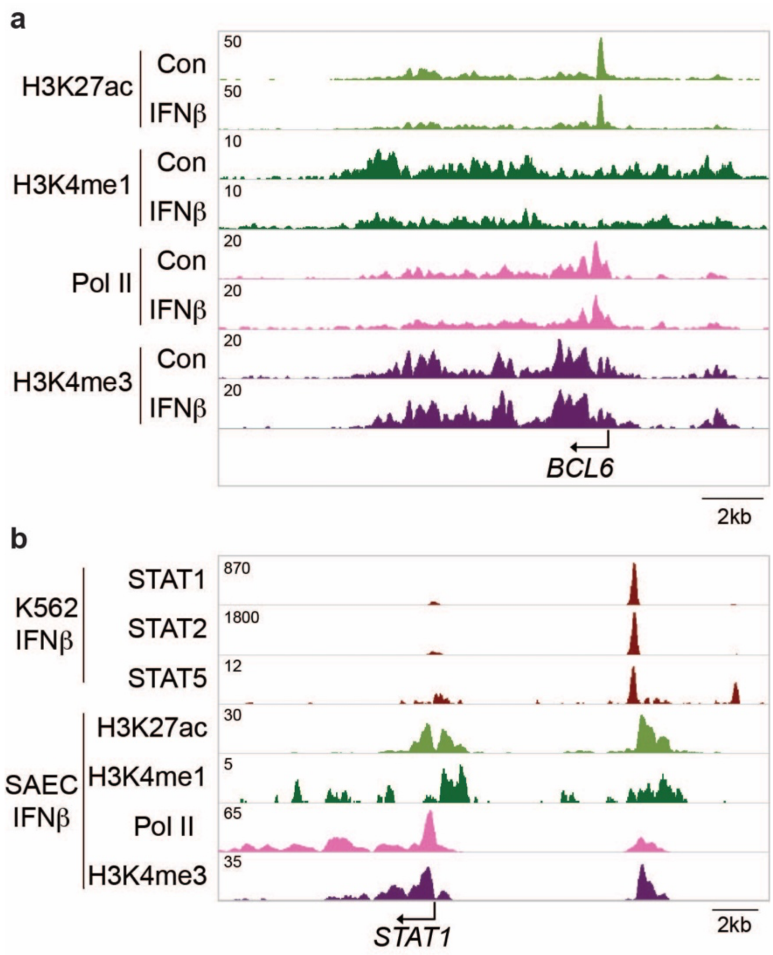

Supplementary Figure 3. Activating histone marks and Pol II loading at control loci. **a.** The *BCL6* severed as ChIP-seq control for histone marks and RNA Pol II. Solid arrows indicate the orientation of genes. **b.** ChIP-seq data for STAT transcription factors, histone marks and RNA Pol II on the *STAT1* locus.

bioRxiv preprint doi: https://doi.org/10.1101/2020.10.04.325415; this version posted November 6, 2020. The copyright holder for this preprint (which was not certified by peer review) is the author/funder. This article is a US Government work. It is not subject to copyright under 17 USC 105 and is also made available for use under a CC0 license.

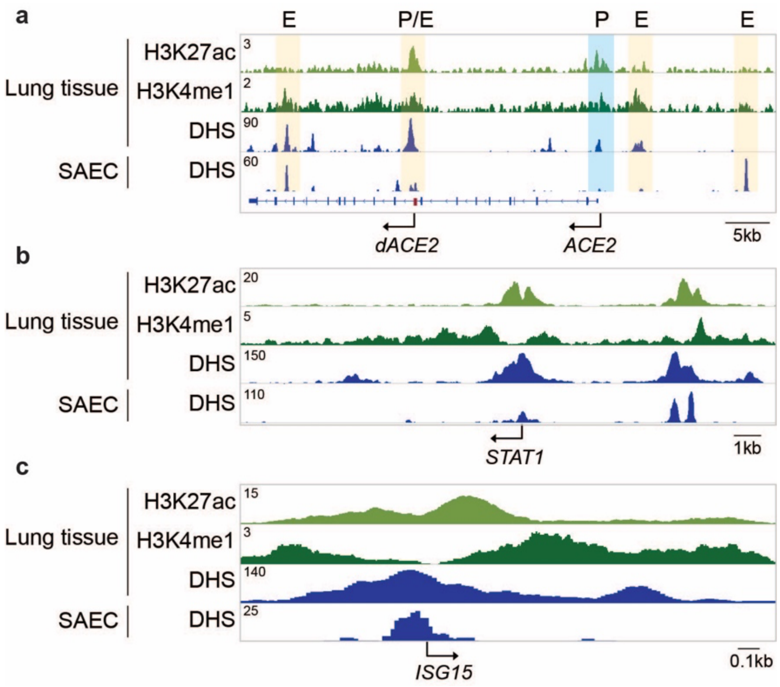

Supplementary Figure 4. Activating histone marks at the *ACE2* locus in lung tissue. ChIP-seq and DHS from human lung tissues and DHS from SAECs display regulatory elements at the *ACE2*, *STAT1* and *ISG15* loci.

bioRxiv preprint doi: https://doi.org/10.1101/2020.10.04.325415; this version posted November 6, 2020. The copyright holder for this preprint (which was not certified by peer review) is the author/funder. This article is a US Government work. It is not subject to copyright under 17 USC 105 and is also made available for use under a CC0 license.

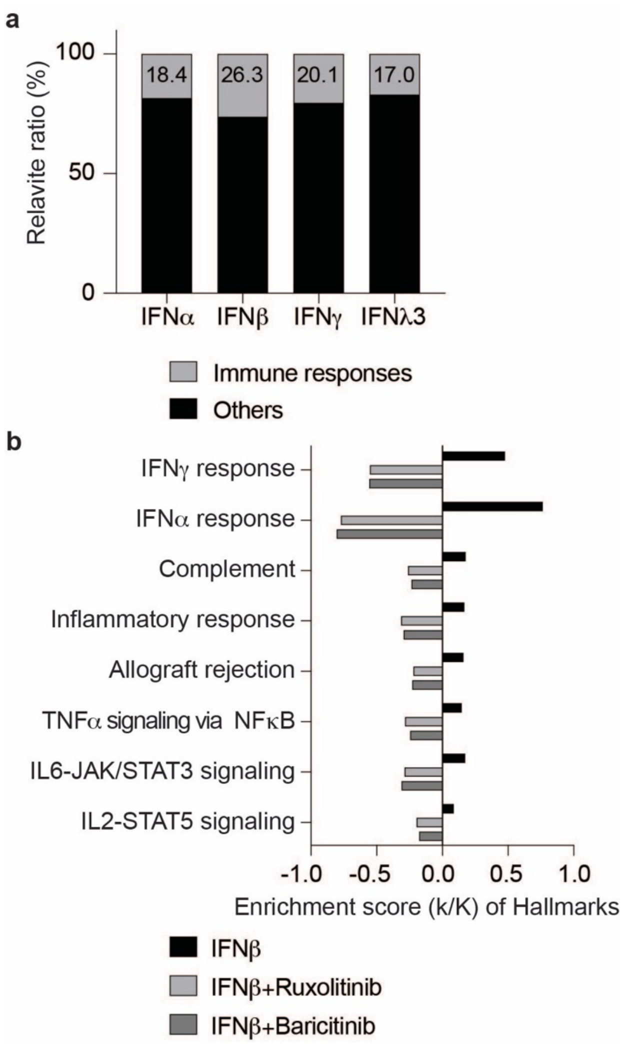

Supplementary Figure 5. Activation of immune response genes by Interferons in human primary airway epithelium (SAEC) is mitigated by JAK inhibitors. a. Genes induced significantly by IFNα, β, γ and λ3 were significantly enriched in Hallmark Gene Sets (FDR q-value < 0.005). Immune response genes accounted for 18.4%, 26.3%, 20.1% and 17% of the upregulated genes. **b.** Expression of immune pathway genes induced by IFNβ was mitigated by the JAK inhibitors Ruxolitinib and Baricitinib.
